# The use of technology enhanced learning in health research capacity development: lessons from a cross country research partnership

**DOI:** 10.1186/s12992-016-0154-z

**Published:** 2016-05-10

**Authors:** E. Byrne, L. Donaldson, L. Manda-Taylor, R. Brugha, A. Matthews, S. MacDonald, V. Mwapasa, M. Petersen, A. Walsh

**Affiliations:** Royal College of Surgeons in Ireland, Dublin, Ireland; Dublin City University, Dublin, Ireland; College of Medicine, University of Malawi, Blantyre, Malawi; Irish Forum for Global Health, Dublin, Ireland; Concern Worldwide, Lilongwe, Malawi

**Keywords:** Research capacity strengthening, Blended learning, e-learning, Partnerships, Malawi, Health systems

## Abstract

**Background:**

With the recognition of the need for research capacity strengthening for advancing health and development, this research capacity article explores the use of technology enhanced learning in the delivery of a collaborative postgraduate blended Master’s degree in Malawi. Two research questions are addressed: (i) Can technology enhanced learning be used to develop health research capacity?, and: (ii) How can learning content be designed that is transferrable across different contexts?

**Methods:**

An explanatory sequential mixed methods design was adopted for the evaluation of technology enhanced learning in the Masters programme. A number of online surveys were administered, student participation in online activities monitored and an independent evaluation of the programme conducted.

**Results:**

Remote collaboration and engagement are paramount in the design of a blended learning programme and support was needed for selecting the most appropriate technical tools. Internet access proved problematic despite developing the content around low bandwidth availability and training was required for students and teachers/trainers on the tools used. Varying degrees of engagement with the tools used was recorded, and the support of a learning technologist was needed to navigate through challenges faced.

**Conclusion:**

Capacity can be built in health research through blended learning programmes. In relation to transferability, the support required institutionally for technology enhanced learning needs to be conceptualised differently from support for face-to-face teaching. Additionally, differences in pedagogical approaches and styles between institutions, as well as existing social norms and values around communication, need to be embedded in the content development if the material is to be used beyond the pilot resource-intensive phase of a project.

## Background

The Commission on Health Research for Development, formed in 1987 and based on a global analysis of health conditions and health research, found that research is essential for health action, but also is needed to contribute new insights and alternative interventions [1]. In its final report, presented at the Nobel Conference in Stockholm (Feb 1990), the Commission presented strategies through which the power of research could be harnessed to improve health outcomes and address health inequities, including the strengthening of expertise in research as “one of the most powerful, cost effective and sustainable means of advancing health and development” [2], p165.

The Council on Health Research for Development (COHRED), which was established in 1993 to promote essential national health research, stated in their Annual Report 2008 that within the last two decades: “there has been a burgeoning of global organisations, partnerships, initiatives and meetings – all focussed on strengthening aspects of health research for development across the globe, and each proposing a different route to this end” [3], p.2. One of the calls for action from the 2004 Mexico Summit (Ministerial Summit on Health Research) was for research capacity strengthening [1]. The First Global Symposium on Health Systems Research held in Montreux (2010) called for ‘a new international society for health systems research, knowledge and innovation’. This symposium was the launch pad for the now highly active Health Systems Global network (http://healthsystemsglobal.org/) and the fourth global health symposium conferences is due to be held in Vancouver in late 2016. There have been numerous other conferences and summits over the last two decades which have emphasised the importance of health research capacity strengthening, but there is recognition that this is undervalued regarding the role it plays in improving equity of health service delivery and advancing human development [4]. Alongside this and similar other calls for health research capacity strengthening are the divergent opinions on what constitutes research capacity strengthening and how this is to be achieved.

In the last two decades, there has been an expansion in the definitions and types of research capacity strengthening in the published literature [5–9]. Previously, there was a perception of research capacity development as funding studies in Lower and Middle Income Countries (LMIC’s) that focused on the individual researcher (technical skills, technology, career paths, peer reviews, publications). Underpinning many of these projects or initiatives was the assumption that the recipients were ‘empty vessels’.

*… that is, the assumption by those who position themselves at the center of some form of knowledge production that there is no knowledge anywhere else, but only empty receptacles waiting to be filled. In other words, to put it bluntly, mistaking one’s own ignorance of what exists elsewhere – knowledge, information systems, practices – for their absence.* [10]*, p.4*

More recent literature relating to individual research capacity strengthening focuses on training models, particularly for MSc and Ph.D programmes. Davies et al. [11] found that scientists in the UK have nearly 1,000 times more opportunities to study for a Ph.D. than do researchers in LMICs and over half of postgraduate qualifications in LMICs were obtained wholly or partially overseas. The traditional Ph.D. training model involves training abroad at an affiliated institution and working with a researcher whilst maintaining linkages to the country where the student originates [12]. The sandwich Ph.D. (The Swedish International Development Cooperation Agency model http://bit.ly/24jLKT3) has become more popular recently, whereby most of the training occurs in the home country, with short periods of time spent abroad for particular courses.

Though individual capacity development is still recognised as valuable, current understanding of capacity development has moved from the focus on the individual to a more multi-levelled approach where individual researchers, research teams and institutions, and the national research structures and environments in which they operate collectively constitute the national research system [13]. Several authors consider three levels of research capacity: i) environmental and network capacity; ii) organisational/institutional capacity, and; iii) individual level capacity [9, 12, 13]. The importance of linking the various levels of capacity strengthening has been stressed [12]. In addition to the three levels, Manabe et al., [12] highlight a foundation level, “local context,” which outlines the need for capacity building to recognise cultural factors, alignment with local and national policies and strategies, trust among development partners, and local ownership. There is also recognition that capacity development goes beyond training and beyond the implicit assumptions of capacity building (where it is assumed that the community does not have any capacity at all to begin with and that the outsider is starting from scratch).

The earlier literature on research capacity strengthening emphasised LMICs’ role in building capacity with researchers at the receiving end, displaying north-south inequities in the process. In recent years, it has been reported that often the Higher Income Countries (HIC) researchers capacity is also enhanced, as they learn from their LMIC colleagues how to deal with different cultural contexts, and how to adapt research methodologies. Here, research capacity strengthening is seen as a two-way process [14]. But knowledge gaps still exist on culture and context in the health research partnership capacity strengthening literature [15–17]. Maher et al. [17] demonstrate the distinct layers of culture within and between different institutions and disciplines and highlight the importance of learning different organisational cultures and structures, although these layers are not explained in detail in their study.

Thus, within the research capacity strengthening literature, there is recognition of the need for a more collaborative approach to research capacity strengthening, for capacity development to be viewed as a two-way process and for training that is adapted to the context and culture in which it is to be used. For this article the definition of health research capacity of the Global Forum for Health Research in 2004 is used.

*Research capacity development is the process by which individuals, organizations and societies develop abilities (individually and collectively) to perform functions effectively, efficiently and in a sustainable manner to define problems, set objectives and priorities, build sustainable institutions and bring solutions to key national problems.*[18]*, p.150*

There is also an expanding debate on the role of technology in teaching and training that is also relevant to strengthening research capacity. The benefits of using web-based tools (commonly defined as online tools and other network resources and technologies) include overcoming temporal/geographic or physical access barriers; providing searchable content and encouraging interactivity; achieving greater student focus, as learners can have greater control over timing and sequence of learning; and obtaining higher retention and improved student satisfaction [19–24]. Pedagogical advantages that are suggested include supporting constructivist approaches to learning (whereby learners construct their understanding and knowledge through experience and reflection) and socialising online learning to a greater extent than previously possible [25]. Web-based tools also offer greater flexibility in the learning process, easier publication and reusing of study content by students and teachers/trainers [26], and; facilitating more active learning and collaborative knowledge building [27].

While recognising the potential of electronic learning and the explosive growth of the Internet as important drivers of education transformation, the challenge for many educational institutions and individuals of poor information technology infrastructure needs to be addressed [28]. Given that it may not be feasible to offer fully online programmes in many parts of the world due to inadequate internet connectivity, low bandwidth and limited computer ownership, blended learning approaches may be the most appropriate [29, 30]. A blended learning programme is a combination of face-to-face instruction with computer-mediated instruction [19].

Technology enhanced learning can also be seen as a means for resource constrained countries to improve access to medical education and overcome a shortage of teachers/trainers [29]. Ellaway and Masters [31] noted that e-learning has become mainstream in medical education (e-learning covering broadly ‘the educational uses of technology’), and the amount of e-learning resources available to an educator has increased dramatically [23]. However, a recent survey of courses in health policy and systems research reported that the use of online education or technology enhanced learning was minimal, both in LMIC and HIC settings [32]. Therefore, although the transformation of medical education through e-learning has started, it needs to be adapted and tested in new areas such as research capacity strengthening; and it needs to be supported to meet the growing demand and the need for greater access to it, especially in the global south where institutional and other resource shortages are greatest.

The information society [33] with the corresponding explosion in technology choices and available information requires changes in how we educate or teach. In transforming education to strengthen health systems Frenk et al. [34] note that there needs to be explicit links made between the education of health professions and the health systems where they will practice, which requires the design of new instructional and institutional strategies. They argue that this requires a move away from "… inward-looking institutional preoccupations to harnessing global flows of educational content, teaching resources, and innovations” [34], p.6. Additionally, they note the need for new competencies to be developed to deal with the explosive increase, not just in the volume of information, but also in the ease of access to it.

Working within this information society requires new skills in aggregating and analysing vast amounts of information and in the extraction and synthesis of knowledge that is necessary to researchers for professional practice [34]. This point is echoed by Ruiz et al. [23] who emphasise that new skills are needed by the educators to transform from traditional teacher to a curator of information and facilitator of learning. “We must embrace, adapt to, and harness technology in order to meet the needs of present and future health professionals” [28], p.439. Just as technology enhanced learning is becoming essential for the health professional and the health systems practitioner, it can play a role in strengthening research capacity in ways that are cost effective and culturally appropriate, through allowing researchers to remain in and develop their skills in LMIC settings and while on-the-job.

This article explores the use of technology enhanced learning in the delivery of a collaborative postgraduate blended Master’s degree in Malawi that focused on research capacity strengthening in community health systems research and addresses two research questions:

I. Can technology enhanced learning be used to develop health research capacity?
II. How can learning content be designed that is transferrable across different contexts?

### MSc in community health systems research

The Community Systems Strengthening for Equitable Maternal, Newborn, and Child Health (COSYST-MNCH) project was funded by Irish Aid/ Higher Education Authority (of Ireland) 2012–2015 as part of the *Programme of Strategic Cooperation between Irish Aid and Higher Education and Research Institutes* [35]. The project was a partnership of experienced development workers, researchers and practitioners in the Royal College of Surgeons in Ireland (RCSI – lead); College of Medicine (CoM), University of Malawi; Concern Worldwide (CWW), Ireland and Malawi, and; Dublin City University (DCU), Ireland. The goal of COSYST-MNCH was to achieve a better understanding of community systems factors underpinning maternal, newborn and child health (MNCH) services in Malawi, focusing on the health dimensions of the first 1000 days of life.

COSYST-MNCH had two components. The first was research case studies of districts and community settings where CWW Malawi was already implementing projects. A central aim was to understand how community systems influence MNCH service utilisation. Inherent in this approach was an understanding that broader intersectoral factors such as hunger, nutrition, and poverty are major determinants of MNCH service utilisation. The second component involved the development and delivery of a Masters in Community Systems Health Research in Malawi. This component offered capacity development opportunities to all the country partners in the form of blended learning – combining new technology enhanced modules and face-to-face training.

Through these two components, COSYST-MNCH aimed to:

- establish an international and cross sectoral research partnership to enable learning by the partners and generate knowledge on and for strengthening community systems for MNCH;
- improve the evidence base on the community systems obstacles and enabling factors underpinning MNCH service utilisation in Malawi within the first 1000 days of life, and;
- enhance capacity amongst key development partners (i) to identify, measure and analyse health problems and services at the community level, using both quantitative and qualitative methods, and/or (ii) in designing and delivering technology enhanced education.

It is within the second component – the blended learning Masters – that, as reported here, a range of e-learning technologies and applications were used with varying success and acceptability.

The MSc in Community Systems Health Research was a blended Masters programme, comprising a modular taught course and research dissertation. The MSc targeted practitioners and students with a background, experience or interest in community development, who wished to develop and/or build on existing expertise in health research. An objective of the partnership – by undertaking the pilot in conjunction with and located in CoM – was to introduce an innovative e-learning educational programme which could be taken over, adapted and accredited through the University of Malawi, once the pilot phase was complete. The MSc was launched in March 2014 in Malawi and was tested and evaluated over the period 2014–2016. RCSI led the project with DCU providing technical and academic support, and academic and logistical input provided by CoM and CWW. The MSc was accredited by RCSI.

The MSc combined technology enhanced online modules with face-to-face sessions delivered in Malawi. Approximately 80 % of the Masters was provided online. The learning technologies used in the programme included Padlet (https://padlet.com/), Twitter (www.twitter.com), Wikispaces (www.wikispaces.com), Google Docs (docs.google.com) and Wordpress (https://wordpress.org) - tools specially selected to enhance learner-content, learner-learner, and learner-instructor communications. In computing terms these web-based tools that were used are not particularly new or novel, but it can be argued, due to their limited application in educational context, that they are new and novel in the field of education. Downes [36] argues that the emergence of the newer web-based tools is a social revolution rather than a technological revolution – a culture referred to by Bryant [25] as the ‘always on’ culture.

The structure and outline of the modules were agreed at a 2013 cross-country workshop that involved all the partners, where there was consensus on the need for a balanced mix of quantitative and qualitative research methods. The MSc comprised six modules, with two delivered in each of 3 semesters in an 18 month period. Each module was taught over a period of 8–9 weeks. The modules covered health systems, community systems, epidemiology and statistics, research methodologies and methods, and measuring health. An orientation module was designed to acclimatise learners to an online learning environment, introduce them to the programme and to some of the tools that would make up their future educational journey. Students were required to complete this non-credit bearing module before commencing the six core modules. Each semester, a one week intensive course was delivered mainly by RCSI and DCU staff with support from CoM, covering the two modules in the forthcoming semester. After the six modules were completed students continued with a research project and thesis write-up over an 18 month period. The teaching materials and supporting activities were hosted in Moodle (https://moodle.org), an online virtual learning platform that is widely used by higher education institutions worldwide, and a CD version of these materials was created to provide offline access to content. Pre-paid internet access cards were arranged for the students, allowing them five free online hours per week, which was deemed to be the amount of time necessary for completion of the weekly online activities. Development workers/practitioners employed by two Non-Governmental Organisations (NGOs) working in development in Malawi comprised the first intake to the MSc (5 students).

The use of web-based tools requires a pedagogical shift on the part of academics - a change from ‘teacher-centred knowledge-transfer’ models to a more active and constructivist approach to problem solving: ‘A new educational culture and mind-set as well as overcoming considerable organisational barriers are important prerequisites’ for this approach [37], p.265. However technology alone will not deliver the educational benefits, which: “only becomes valuable in education if learners and teachers can do something useful with it” [38], p.24.

Improving the technical competencies of students, teachers/trainers and the wider programme team across institutions was a key aim of the project. Professional development for educators lowers technology anxiety and discomfort and therefore encourages adoption of learning technologies [39]. Formal information and learning technology training sessions were conducted in Ireland and Malawi to share plans, experiences and best practices across institutions, and to extend technology enhanced learning competence beyond project members. These were supplemented by tailored, one-on-one training for individual teachers/trainers, delivered by a learning technologist, as they initially developed online content.

The Masters programme, by seeking to directly build the research capacity of NGO staff – rather than just involving them in the collecting of data for the research element of the project – aimed to achieve a more equitable partnership between academics and NGOs, by enabling NGO staff to work towards academic awards, while also facilitating the completion of the larger project. It also offered experienced RCSI staff, most of whom had limited or little experience of technology enchanced teaching methods and approaches, opportunities to spend time on creating content and reflecting on modes of delivery in new and often challenging ways. The benefits of technology enhanced learning were not limited to the development of online content and extended to the design and delivery of existing face-to-face education programmes in RCSI and CoM.

## Methods

An explanatory sequential mixed methods design [40] was adopted for the evaluation of the technology enhanced learning in the MSc programme. The COSYST-MNCH project was granted ethical approval by the COM research ethics committee (March 2104 P.08/13/1443). Audits and end of course evaluation of RCSI teaching programmes are not required by RCSI research ethics commitee. The technical competencies of the teaching/training staff were assessed at an early stage in phase 1, and the skill and knowledge set ranged from basic to more advanced technical skills and knowledge. This assessment helped shape technology decisions and set the level and types of support provided. Phase 1 included a quantitative online survey of students before the commencement of the taught course modules as well as online surveys at the end of each module (see Table 1). The baseline student survey (Kwiksurvey - https://kwiksurveys.com/), administered before content was designed, assessed available technical systems and infrastructure and the digital literacy of students. A similar survey was conducted for teachers/trainers involved in the development and delivery of the programme (see Table 1). The findings were used to create the digital literacy training inputs for teachers/trainers and a mandatory orientation online module for students was developed. This complemented and reinforced face-to-face training that was delivered to them in Malawi at the start of the taught programme.

**Table 1:** Data collection methods

Data collection instrument	Number	Purpose
Phase 1 (on-line surveys & review of collaborative forums)		
Institutional baseline survey	4 (RCSI, DCU, COM and CWW)	To determine infrastructural capabilities of the institutions in terms of development and delivery of learning content.
Faculty baseline survey	8	To determine technological capabilities and experience of faculty.
Student baseline survey	5	To determine infrastructural support and technological capabilities of the students.
Online survey (end of each semester)	3 occasions	To monitor the content and technology used in the delivery of the six taught modules.
Interaction in collaborative forums	Forums included: • Moodle discussion forums • Padlet walls • Wikis • Twitter	To review level of participation in online activities across all six taught modules.
Phase 2 (independent evaluation)		
Semi-structured interviews	8 Teacher/trainers (2 DCU; 6 RCSI)	To understand faculty experience, challenges and benefits and motivation with respect to the MSc.
Semi-structured interviews	3 students	To understand students' motivation and experience, challenges and benefits with respect to the MSc.

The online survey that was administered to students at the conclusion of each semester assessed their familiarity and degree of confidence in using various technologies, the balance between interactions and exercises with delivered content, the appropriateness of the course material, and student satisfaction with instructors and delivery of the module. All surveys contained closed and open-ended questions including a request for suggestions to improve course materials.

The virtual learning platform (Moodle) provided quantified data on students’ participation in the modules, which complemented learning logs of completed exercises and interactions (see Table 1). Online student contributions to Discussion Forums, Padlet walls, Wikis, and Twitter, were recorded and analysed. Descriptive statistics for Phase 1 were calculated using Microsoft Excel.

Phase 2 (see Table 1) of the study involved the recruitment of an independent evaluator with the aim of achieving a better understanding of the motivation for, and experiences of, participating in the MSc, as well as challenges and opportunities faced. The number of teachers/trainers directly participating in the teaching was 8 (6 RCSI and 2 DCU) and the number of students was 5 for Modules 1 and 2, and 4 for the remaining modules (1 student withdrew from the programme). Semi-structured interviews by the independent reviewer were conducted face-to-face with teachers/trainers and via Skype (due to geographical locations) with the students. Participants were provided with an information letter describing the interview process, and how the data would subsequently be used, before the interview. Before commencement of the interviews, participants were asked to confirm their informed verbal consent to participate in the study. The interview audio was transcribed verbatim, and the qualitative data was thematically analysed using a semi-inductive iterative coding approach using QDA Miner Lite (http://qda-miner-lite.software.informer.com/).

## Results

Based on the findings from the online evaluations of the modules (phase 1) and the results of the independent evaluation (phase 2) the combined results emerged as four main themes: Design; Information technology choices and support; Human connection, and; Institutional support.

### Design

The ability to remotely collaborate and communicate was of paramount importance for teachers/trainers and students. Differing needs and capacities of the teachers/trainers and students, limitations of Malawian technical infrastructure and the technology enhanced learning strategies of the different higher education institutions required consideration in the planning of the programme. Ultimately, the strategy that guided the development process was to:

- design a simple, streamlined process to develop pedagogically appropriate course content across institutions;
- use technology to create feasible, innovative and collaborative learning experiences; and
- improve the technical competencies of teachers/trainers and students whilst minimising technical anxiety.

Trainers/teachers are faced with a plethora of technology choices when considering implementing a blended learning solution. Such a wide selection creates difficulty in choosing the optimal tools for developing technology enhanced content and may contribute to making ill-formed learning design choices [41]. As technology choice is an ever-changing environment these options needed to be continually updated - a tool was developed by the learning technologist to assist with this.

Making the technology available is not sufficient, as training and ongoing support is vital to increase educator confidence and to encourage adoption and integration of learning technologies [42]. Thus the course design was an iterative process involving initial development by teachers/trainers, technology enhancement discussions with the learning technologist and subsequent development of course files. The orientation model included an overview of the tools and how to use them and was a compulsory module for all students. Teachers/trainers were trained on these tools as part of content development. There was also a staged introduction in the modules of the various tools, with Module 1 using only three tools and the other modules expanding on these.

In the module evaluations one of the questions included in the students' questionnaire was: ‘Was there an appropriate balance of content and activities?’ Across the six modules 2 of the students felt that there was an appropriate balance and 2 did not. What was interesting was that 1 student felt there was not the right balance in all the modules regardless of the fact that one of the modules only had 2 activities and another had 28. A possible explanation for this was though the face-to-face teaching that took place was appreciated one student reported disappointment with the low level of face-to-face teaching and perhaps thus felt that the interactions online did not meet their expectations regardless of the level of online interaction built into the module.

*My …expectations was that we might be meeting quite often … but … what came out to be was that … we were occasionally meeting, and maybe a lot of work was given for us to go through when we’re back home. I thought maybe we could be meeting once a month or thereabouts, but we are meeting in, I think in three months or four months.* (Independent evaluation Participant 8)

This, however, was compensated for by the fact that the students were able to undertake the MSc whilst still working and staying at home. The students reported a number of benefits that they perceived to be offered by the online learning model – frequently these focused around the flexibility that was provided by online learning to allow them to work, and manage their family responsibilities:

*It’s very useful, because I was able to manage my family, I was able to manage my responsibilities because I was working, and I was getting something from my work, at the same time I was doing school, so I was upgrading academically, but I was also supporting, the usual support that I provide to my family …* (Independent evaluation Participant 3)

Additionally, students spoke of the usefulness of the online learning model, which enabled them to undertake education without having to commit the time to travel and be present in class:

*… if it was not online, I would be required to taking class daily, yeah, so it has given me a chance … to do so many things at once … it was not going to be possible if … it required me to go in class.* (Independent evaluation Participant 7)

### Information technology choices and support

Technical infrastructure and systems to support blended learning are improving globally, although in Malawi internet penetration is still very low, at 4.4 % (http://www.internetworldstats.com/africa.htm). Students living outside the main cities in Malawi continue to have significant infrastructural issues regarding Internet access. The technology choices made for this MSc were predicated on the personal expertise and experience of the project team with an aim of supporting the overall learning outcomes of the programme. It was important the MSc utilised technologies that functioned with existing infrastructure across the three higher education institutions and built on current expertise and familiarity, as that fostered engagement and collaboration between instructors and students.

Moodle was chosen as the virtual learning platform as all three higher education institutions were already using it. Articulate Studio 13 (https://www.articulate.com), a rapid e-learning development tool, was selected to develop course materials because teachers/trainers were familiar with the Microsoft PowerPoint environment. Working with Articulate Studio 13 proved challenging at times, as the software was not found to be as robust as hoped and problems with saving recordings and transferring files between teachers/trainers and the learning technologist arose during development. This is especially problematic given that teachers/trainers were based in and had to share files electronically across two separate institutions in Ireland. Many hours of potential content development were consumed in overcoming these challenges, which contributed to the demotivation of staff at different times in the development process. However, strategies were devised to overcome these difficulties and teachers/trainers felt that through the partnership they had developed their technical skills.

*Articulate was the main one of course, because … that was the main, main vehicle for delivering the content of the Masters. So, we learned an awful lot about that and [other staff name] coached us through how to use Articulate, and how to record in Articulate and all of that, yeah.* (Independent evaluation Participant 2)

**Fig. 1:**
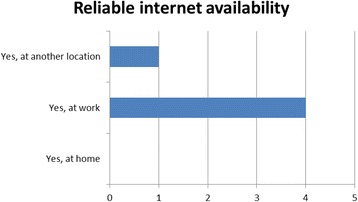
Number of students indicating reliable internet connectivity at baseline

**Fig. 2:**
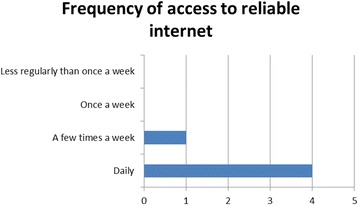
Number of students indicating frequency of access to reliable internet connectivity at baseline

The baseline resource survey indicated that there was adequate internet bandwidth to enable students to access course materials online, as Figs. 1 and 2 indicate. However, the reality in delivering learning online proved to be different. From the outset the COSYST project team were aware that the infrastructure in Malawi was likely to be weak for online delivery of medium to high bandwidth content, such as streaming videos or synchronous activities. However, it only came to attention in the first face-to-face session that students did not have reliable access to the internet. Students access was restricted to office hours, and they had no access when they were ‘in the field’ (i.e. running community-based projects) or at home. Perhaps students felt that if they had raised this in the baseline, they might not have been selected for the course. Alternatively, the students had genuinely felt that the limited access they had to the internet was adequate for an MSc until they realised what was practically required.

To circumvent the problems with access to the internet Compact Discs (CDs) had to be burned and distributed to students before commencement of the modules. The CDs were then used as the primary method for content consumption and potentially impacted on the effectiveness of collaborative online activities. Dongles and monthly internet airtime also had to be purchased for each student. In the independent evaluation of the first two modules most of the students raised internet access as the primary challenge they had in doing the modules.

*… it was difficult on the other part because of the internet here in our country … it’s not reliable, yeah, most of the time the electricity is off, and the internet is not reliable, so I don’t know how we can, I can say it could be improved, maybe if the internet in our country had improved … it would make life easier on the assignments … because if it was easier to go, if we had the means, to go on the Moodle to check … sometimes … we would miss the deadlines because of that, because I had no access to the internet, so it was making life difficult on that part.* (Independent evaluation Participant 7)

Intermittent access to low bandwidth internet also had implications for the types of  technologies that could be used. The access to videos was limited due to poor connectivity as was synchronous video conferencing. Two ‘live’ synchronous text chats were possible and proved an effective medium to communicate with the students in real time. Students came together online with the teacher/trainer at a set date and time via the Chat facility in Moodle, which provided the opportunity for students to ask questions and generally discuss how the modules were going directly with the teachers/trainers and the other students. The immediacy of the medium was praised by the students following the sessions despite some dropouts and reconnections due to poor Internet connectivity.

One of the learning outcomes and part of the graduate profile for the MSc was: ‘Ability to utilise a technology enhanced learning platform for supporting learning.’ Student skills in the use of a number of the technologies improved, as shown in the evaluations at the end of each semester, which indicates an increase in competencies in the technologies used. All students strongly agreed with the objective of developing their technical skills as part of the programme in the baseline survey, though some students did request training to re-hone competencies. This was offered during the orientation module and again at critical  points during the programme. In the independent evaluation all students agreed that they had achieved this objective and that due to their involvement in the MSc, their level of ability in using technology had increased. However, with the provision of airtime ceasing after the modules were completed, the continued usage of these tools may be problematic.

*… my level of experience, I think, I have gone up since I started this course … before that, I didn’t have much knowledge on the information technology, but now I’ve learned … a lot of technologies … I’ve learned about WordPress, I’ve learned about … different methods of the technologies … for example using the Facebook … I have learned to use the Padlet … before this course, I didn’t know how to use them, but I’m glad now that, yeah, I can also use these things. (Independent evaluation Participant 7)*

**Fig. 3:**
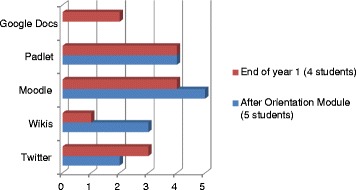
Number of students who perceived that they were competent in the use of various technologies

There was an interesting reduction in students perception of their perceived competency in wikis as the MSc continued (Fig. 3). This may be attributed to wikis being only used as part of Modules 1 and 2 and hence skills may have declined over time due to lack of practice with this particular tool. Google Docs was not used until the second semester and was not included in the orientation module so competency in this area was only recorded at the end of year 1. Difficulties in setting up accounts in Twitter, which were initially blocked due to the students having Irish email addresses whilst setting up accounts in Malawi, were overcome. However, the take up and usage of Twitter outside of course requirements still appears limited. Twitter requires the user to be conversant with twitter lingo, such as # hash tags, requires the user to know who to follow and what is trending; and limits the user to 140 characters to express his or her thoughts. These reasons, and a lack of use of Twitter in social communications in Malawi, may have contributed to it not being used in an academic link between Africa and Ireland, despite claims to  being the number one tool for learning for the last 7 years (http://c4lpt.co.uk/directory/top-100-tools/).

### Human connection

The level of participation and engagement varied across the modules in terms of the number of activities/exercises/interactions that were designed within each module, the level of engagement and interaction of the students, and the level of engagement and interaction of the teachers/trainers. The learning activities were designed to be stimulating and collaborative. In the independent evaluation the students spoke of the value of collaboration and working as a group:

*… in a group is better, not yourself … critical is just to be able to share some of the things … with the group. We realised that we are all strong because we are able to travel with other people, so in a group … it’s easier in a group than yourself.* (Independent evaluation Participant 3)

However, engagement on the part of the students online was patchy during delivery of the six modules. It emerged in the module evaluation at the end of semester two that most of the students had not realised the level of engagement that would be expected of them. In practice, some activities and technologies were better received than others, for example Padlet wall postings. This was possibly due to the simplicity of use; whereas Twitter post exercises saw less activity, possibly due to it requiring relatively greater or more frequent engagement. The provision of content on CDs meant that students could engage with the content offline, although engagement online was needed to complete most learning activities. Formal assessment of these online activities occurred in the later modules as teachers/trainers wanted to ensure that internet access problems were resolved and students’ confidence developed through practice in the earlier modules before assessing contributions formally. Levels of participation increased dramatically when incorporated into formal or summative assessments and challenges in accessing the internet for this involvement were overcome.

Interestingly, it emerged in the independent evaluation (and previously had been assumed was happening by some of the teachers/trainers) that a number of tools, including online tools,  were used by students to communicate with each other -  the frequency with which these were used was not mentioned:

*… we are using WhatsApp, we are using Skype … we’re using Dropbox to share information, yeah, like we had an account in Dropbox where everyone can post in something, and can link easily.* (Independent evaluation Participant 3)

In the face of difficulties in accessing the internet, more basic forms of communication were also used, such as mobile phone calls and emails. Students also had arranged meetings between themselves to come together to work – not at the behest of the teachers/trainers, and all mentioned the value of collaboration:

*It was far easier with the other students because most of them we could just use phones at any time, and when you want to meet, we’d just be able to circulate emails, because we are, we had … an email which you could just circulate … we did far, much better with the other students. …we used to make our own meetings, we would arrange to, to meet, though we were staying in different districts …* (Independent evaluation Participant 8)

Students also acknowledged the role of good communications with the teachers/trainers for getting support and feedback:

*… when we are stranded, COSYST team were there for us, just to check whether we are within, or we are still struggling, so I like that kind of checking from the, from the COSYST team, it helped us so much.* (Independent evaluation Participant 3)

It was evident from participants’ responses in the evaluation report that the two groups of stakeholders – i.e. teachers/trainers and students – communicated well within their own respective circles. Teachers/trainers affirmed the usefulness of interactions with each other, and valued the support that was available, for example in the form of the learning technologist. Simultaneously, the students expressed a preference for working together and organised sessions in parallel to those outlined in the course design. However, at the stage where communications between students and teachers/trainers might be expected to occur through the online forums this was not the case. Here, the expectations and culture of learning among the group of students themselves may have been a limiting factor. Teachers/trainers reported that students were clearly able to use the necessary communications channels available to them if there had been a problem, but did not proactively seek information or engage in online activities unless required to do so. In the online activities discussion was also one-way, that is in response to a question or scenario posted by teachers/trainers and rarely did a discussion occur between the students or was self-initiated by students.

### Institutional support

In the independent evaluation,  teachers/trainers acknowledged the support of the RCSI’s accreditation and qualifications committee, and other staff who facilitated the process of making the business case for the MSc to senior management and other technicalities of accreditation:

*… we had a huge level of support from the accreditation qualifications committee … I think it’s important we acknowledge that … the accreditation document, very complex, because we were moving from, if you like, terrestrial to distance learning from which, you know, because the pedagogy is different, the whole way you structure, you design the course. We didn’t get it perfect, I think we, we had some flexibility, we should perhaps have built in some more flexibility along the way, the devil is in the detail with marks and standards, and a huge amount of credit should go to [other staff name] in particular, for her oversight of the whole process.* (Independent evaluation Participant 6)

The provision of adequate technical support from the learning technologist who had a high degree of experience in using the technological tools was recognised by many as a key enabler. The learning technologist was employed specifically for the project on a part-time basis.

*… to run, to develop, and to deliver a Masters like this, you need a learning technologist to help you along the way … I think that’s important … they might just say you need a learning technologist for the early stages of it, until you train the staff, but my experience would be … everyone should, should actually have a dedicated learning technologist whether [other staff name] is 50 %, probably in fact definitely has worked more than a part-time job on it, but I think that definitely worked well.* (Independent evaluation Participant 2)

The fears of teachers/trainers with little experience of technology enhanced learning were mitigated due to the employment and support provided by the learning technologist. For example, one respondent had anticipated increased workload, which was then managed through the provision of assistance by the learning technologist.

*… I suppose a benefit, I didn’t realise how much [other staff name] would be doing … at the outset, so … didn’t know if I would be doing all, embedding all the quizzes and stuff like that, so I suppose it was a relief, when it was just me preparing the material, send it on to [other staff name].* (Independent evaluation Participant 4)

It was also evident that the workload involved in preparing course content was in excess of some teachers/trainers original expectations. That notwithstanding, it was also acknowledged that the time spent on content development could also favourably impact subsequent reuse of the content  and teaching practice.

*[Speaking of time commitment required.] No, not at all, so it was a lot more, and I think from talking to my colleagues, everybody says that, like, a lot more, maybe two or three times more than I thought … I think it’s until you actually sit down and do it yourself that you realise that it’s going to take a lot, lot longer, but it makes you think through things a lot more, and as I say being more creative, and have less of this kind of didactic teaching style, so it’s much more about facilitation and interaction.* (Independent evaluation Participant 2)

One teacher/trainer reported that the high workload needed to deliver content to only a small number of students, and without a clear plan for what the materials would be used for in future, or indeed whether they would actually be used again at all, had led to significant demotivation.

*… we knew from the start, this is only going to be for initially five students, four students,... so you knew it was, you had to put in a lot of work, for … very few students, so [it] wasn’t very clear what you can do with it then afterwards, are we going to use it again … it wasn’t very motivational for me, and the other people here, because you put in a lot of work, and then nothing. Nothing’s done with it.* (Independent evaluation Participant 10)

At an institutional level, an additional observation that was made was the fact that the Malawian academic staff appeared not to be interested in taking part in teaching/training, possibly stemming  from a lack of incentives to be involved or more likely the lack of recognition of time spent by CoM staff on the MSc by CoM management as the degree was RCSI accredited.

*Faculty of College of Medicine didn’t get on-board at all, which was interesting. I think they thought all of us were getting paid for doing it, and they weren’t being paid. I presume that was probably where it came from, and if we were to do it again, we’d probably have to pay for additional hour services of these staff if we had to bring them on-board, but then that would cause problems here when you’re asking people to do it for free…* (Independent evaluation Participant 9)

## Discussion

There are well-known challenges in designing a blended learning educational programme, and attempts were made to address these proactively as far as possible, for example being cognisant of  information technology infrastructure; student collaboration and engagement, and; contextually relevant content.

From the outset it was recognised that the availability of technical infrastructure was a fundamental component for delivering online learning [29]. At the design stage, content was developed using tools and material that required low bandwidth. As the difficulties in practice came to light, dongles and internet access cards were purchased and delivered to students. Additionally, the content was also supplied to students on CD and couriered to students in Malawi. However, this solution was resource intensive – both regarding finances and staff time. Though students were quite happy at the end of the taught modules in relation to access, this ‘workaround’ would be extremely expensive if the student group was large.

People do not contribute to online communities from a sense of altruism; rather they hope that they will get information in return, acquire increased recognition and make a difference or gain a sense of community [43]. However, social interaction is a critical part of learning [44] and can be sometimes challenging in asynchronous learning programmes [45]. The lack of a sense of community and a lack of immediacy in feedback can contribute to a sense of isolation for learners – more so if some learners feel more isolated or have greater access problems than others [46]. Emerging technologies can be used as a lever to support a collaborative and effective online learning environment leading to better student outcomes and more satisfied students and teachers [47].

During this MSc, lack of social interaction was a greater challenge than expected for some students who indicated a preference for more person contact hours, which would allow for easier group sharing, if given the choice. This ambivalence about distance learning and preference for face-to-face learning may be attributable to the dedicated time away from work that is often arranged for  employees attending face-to-face courses, but not given for employees taking online courses. However, some students recognised that more contact time would have meant periods away from work and would have prevented them from doing the MSc. Collaboration between students did take place in a parallel forum indicating that students felt the need for peer support in a protected environment that was not monitored by teachers/trainers. The collaboration between teachers/trainers and students via online discussions and other tools was a challenge and may be partly attributable to cultural factors, including the norms and standards in Malawi around communication between teachers/trainers and students and less because of technical difficulties communicating online. This illustrates the importance of taking the pedagogical culture of different institutions and the social culture of society into account when designing cross-institutional content.

The content of the modules was also adapted to/for Malawi through the input of the partners, enabled by both HIC higher education partners – RCSI and DCU –having experience of conducting research in Malawi. CWW provided local data and information on local practical examples of community systems strengthening issues; material from the COSYST-MNCH research component of the project was integrated into the last two modules, and teaching content from modules in CoM was also used. Additionally, discussions, summaries and references to Malawian policies, documents and journal articles were included and some CoM and other Malawian academics were involved in the delivery of some of the modules, during the face-to-face teaching. However, at this stage in the project it is unclear whether any of the content will be used by the Malawian partner after the end of the project, although academic staff at CoM have already introduced technology enhanced learning into their routine work.

The MSc has increased the research capacity of the students who completed the taught course programme, as demonstrated in their performance in the academic assessments, and therefore this case study indicates the potential of blended learning programmes in research capacity strengthening. However, transferability challenges remain that are mainly due to the nature of information technology support in a blended learning environment, and the culture of learning differences between institutions.

With a general drive by institutions around the world to apply technology enhanced learning approaches, the question will not be on whether to use this approach, but rather about which choices to make. This lack of choice is not unusual as an institution rarely chooses innovation freely [48], but it is rather determined by “… events, trends, pressures, opportunities, or restrictions in the international or national arena” [49]. However within these institutions there is the need to recognise that designing a blended learning programme is not as simple as just combining online elements with a face-to-face session [50] but that it should result in the reconceptualisation of teaching and learning activities [20].

This reconceptualisation requires support from skilled personnel in technology enhanced learning [29] – pedagogically trained as well as technically competent. Such skill sets  are not commonplace in an information technology  department and require specific support systems and structures to be built alongside the introduction of blended and on-line learning resource development. The MSc content would not have been developed by the teachers/trainers without the support of the project hired learning technologist.

Additionally, contextualising content is also about adapting learning and teaching approaches and styles.

*“… successful ALN (asynchronous learning networks) learning is not only dependent on optimal uses of available technologies, teachers’ pedagogical content knowledge, and students’ motivation level, it is also dependent on the cultural (mental) representations learners and teachers bring to the learning situation.” *[51]

As Zhao et al. concluded: “*distance education in essence is still education […] The factors found to have an impact on the effectiveness of distance education are also factors that would affect the effectiveness of face-to-face education”* [52], p.1865. The possible differences in formal and informal social norms and institutional rules on collaboration between teachers/trainers in two Irish and one Malawian higher education institution may have been a contributing factor to students developing their parallel peer support network. This may also  have impacted the level of self-initiated online discussion in the shared collaborative spaces that were designed online as part of the modules.

The implications of differences in the expectations of technical support and the norms and rules on student-teacher/trainer collaboration are just two examples of many other potential differences between institutions in an educational partnership. These differences  indicate the need to look at technology enhanced learning from a socio-technical perspective. Technology enhanced learning innovation should be studied as “a combination of technical/rational and institutional action” [49]. These social, cultural, or cognitive forces are located within and beyond an organisational setting and in many cases drive the overall institutional performance [49].

Though we recognised that education is in essence contextual, we changed the content of material without a fuller understanding of the ‘culture of learning’ of the institution/country in which it was to be placed. Simply changing content to be more Malawi focused did not take into account the way in which students are accustomed to learn and interact in Malawi [53]. A pedagogically driven approach to technology enhanced learning requires an understanding of the differences in pedagogy from the outset. Either strategies to change learning styles and other educational differences need to be embedded in the programme or content needs to be adapted to the pedagogical context.

Technology enhanced innovation and its context are so entangled that it would be an oversimplification to see the technology as the content and the society as the context [54]. Such a simplification makes it difficult to understand the multifaceted processes in which technology and humans take part to form socio-technical entities. Content and context are intertwined and impact one another – a concept referred to as ‘duality’ [55]. When studying change and contextualising technology enhanced learning, one should not only consider technology enhanced learning as a different medium for delivering content but rather as an evolution in the networks of and relationships between organisations and people within which these innovations will play a role.

As Du Plooy [56], concerning Information Technology  innovations notes, such changes need to be ‘cultivated’ – developed for the human environment within which the innovation/change is to be implemented. Similar to Manabe et al. [12], Du Plooy [56] describes the human environment as including the social context of the organisation itself; the social context of the groups within the organisation; the social context of the tasks performed and the technologies used to perform them, and; the social context of the broader environment within which the organisations are positioned. It is against this broader contextual approach that a programme needs to be adapted and designed if it is to be transferrable and sustained.

## Conclusion

Overall, teachers/trainers and students learned a lot from the design, development and running of the Masters programme. The type, mode, and frequency of communication that is most suitable and convenient to all partners needs to be continuously updated and negotiated as skills and infrastructure change. There is certainly a potential for Web 2.0 technologies to create more dynamic and creative ways of collaboration within partnerships. Given the recent developments in Web 2.0 technologies and the rise of open and distance learning and e‐learning, teaching and learning have been transformed mostly based on how we can communicate with one another.

The COSYST-MNCH project has been a rewarding experience for those involved in it, in that it encompassed and required successfully overcoming many challenges. The evaluations of the project to date indicated some mixed feelings on the part of the students towards their online learning experience. All students agreed or strongly agreed that they were well supported by their teachers/trainers, but opinions varied as to whether online learning was the right approach for them, which may be a reflection of their particular circumstances in that all were working in community settings where even access to electricity was at times a challenge.

Concerning our two research questions, capacity can be built in health research through blended learning programmes. Three out of the original five students completed the six modules and are conducting their research projects, on the basis of which they will write and submit dissertations for the award of the MSc. Students and teachers/trainers have reported strengthening in their technological capacities. Regarding transferability the support required institutionally for technology enhanced learning needs to be conceptualised differently from support for face-to-face teaching, for example the support of a learning technologist is an essential resource. Additionally, differences in pedagogical approaches and styles between institutions, as well as existing social norms and values around communication, need to be considered when developing content if the material developed is to be used beyond the pilot resource-intensive phase of a project.

## Abbreviations

CDs: Compact Discs (CD ROM)
COHRED: Commission On Health Research for Development
CoM: College of Medicine, University of Malawi, Malawi
COSYST-MNCH: Community Systems Strengthening for Equitable Maternal, Newborn and Child Health
CWW: Concern Worldwide Malawi
DCU: Dublin City University, Ireland
HICs: Higher Income Countries
LMICs: Lower and Middle Income Countries
MSc: Masters in Science
NGOs: Non-Governmental Organisations
RCSI: Royal College of Surgeons in Ireland

## References

[1] Nuyens Y. No Development Without Research. A challenge for research capacity strengthening. . Global Forum for Health Research. Accessed January 8th 2016 at http://bit.ly/1Wt5MYx, 2005.

[2] White F (2002). Capacity-building for health research in developing countries: A manager’s approach. Pan Am J Public Health.

[3] Council on Health Research for Development (COHRED). Supporting national health research systems in low and middle income countries. Making health research work… for everyone. Council on Health Research for Development (COHRED), Switzerland, 2009. At http://bit.ly/2460xUE. Accessed Apr 2016.

[4] Council on Health Research for Development (COHRED). Beyond Aid. Research and Innovation as key drivers for Health, Equity and Development. COHRED. Accessed at www.bit.ly/1VMm8JT on 7th Jan 2016., 2012.

[5] Addullah MS. Research Capacity strengthening – creating demand for research in Kenya. Council on Health Research for Development (COHRED) Learning Brief. 2001;2001/2.

[6] Bates I, Taegtmeyer M, Squire SB, Ansong D, Nhlema-Simwaka B, Baba A (2011). Indicators of sustainable capacity building for health research: analysis of four African case studies. Health Res Policy Sys / BioMed Central.

[7] Bennett S, Corluka A, Doherty J, Tangcharoensathien V (2012). Approaches to developing the capacity of health policy analysis institutes: a comparative case study. Health Res Policy Sys / BioMed Central.

[8] Vasquez EE, Hirsch JS, le Giang M, Parker RG (2013). Rethinking health research capacity strengthening. Glob Public Health.

[9] Nuyens Y (2007). 10 best resources for … health research capacity strengthening. Health Policy Plan.

[10] Suchman LA (2002). Practice-based Design of Information Systems: Notes from the hyper-developed world. Inf Soc.

[11] Davies C, Beattie P, Renshaw M (2000). Out of Africa: Training collaboration and malaria research. Parasitol Today.

[12] Manabe YC, Katabira E, Brough RL, Coutinho AG, Sewankambo N, Merry C (2011). Developing independent investigators for clinical research relevant for Africa. Health Res Policy Sys / BioMed Central.

[13] Lansang MA, Dennis R (2004). Building capacity in health research in the developing world. Bull World Health Organ.

[14] Jentsch B, Pilley C (2003). Research relationships between the South and the North: Cinderella and the ugly sisters?. Soc Sci Med.

[15] Crane J (2010). Adverse events and placebo effects: African scientists, HIV, and ethics in the ‘global health sciences’. Soc Stud Sci.

[16] Martin-McDonald K, McCarthy A (2008). ‘Marking’ the white terrain in indigenous health research: literature review. J Adv Nurs.

[17] Maher J, Sicchia S, Stein LG (2003). Learning the culture of partnership: A case study in collaboration between a Canadian university and its Costa Rican partner. Can J Dev Stud.

[18] Global Forum for Health Research. The 10/90 Report on Health Research, 2003-2004. At http://bit.ly/1VWBNZS. Accessed 8 Jan 2016.

[19] Bonk C, Graham C (2006). The handbook of blended learning: Global perspectives, local designs.

[20] Garrison D, Kanuka H (2004). Blended Learning: Uncovering Its Transformative Potential in Higher Education. Int High Educ.

[21] Lewin LO, Singh M, Bateman BL, Glover BP (2009). Improving education in primary care: development of an online curriculum using the blended learning model. BMC Med Educ.

[22] Fieschi M, Soula G, Giorgi R, Gouvernet J, Fieschi D, Botti G (2002). Experimenting with new paradigms for medical education and the emergence of a distance learning degree using the internet: teaching evidence-based medicine. Med Inf Int Med.

[23] Ruiz J, Mintzer M, Leipzig R (2006). The Impact of E-Learning in Medical Education. Acad Med.

[24] Cheston CC, Flickinger TE, Chisolm MS (2013). Social Media Use in Medical Education: A Systematic Review. Acad Med.

[25] Bryant L (2007). Emerging trends in social software for education. Emerg Technol Learn.

[26] Guntram G, Salzburg Research, EduMedia Group. Open Educational Practices and Resources. OLCOS Roadmap 2012. Salzburg. At http://bit.ly/1Svs9Ln. Accessed 8 Jan 2016.

[27] Mejias U (2006). Teaching social software with social software. Innovate: J Online Educ.

[28] Robin BR, McNeil SG, Cook DA, Agarwal KL, Singhal GR (2011). Preparing for the Changing Role of Instructional Technologies in Medical Education. Acad Med.

[29] Frehywot S, Vovides Y, Talib Z, Mikhail N, Ross H, Wohltjen H (2013). E-learning in medical education in resource constrained low- and middle-income countries. Human Resour Health.

[30] Boitshwarelo B (2009). Exploring Blended Learning for Science Teacher. Professional Development in an African Context. Int Rev Res Open Distance Learn.

[31] Ellaway R, Masters K (2008). AMEE Guide 32: e-Learning in medical education Part 1: Learning, teaching and assessment. Med Teach.

[32] Tancred T, Schleiff M, Peters DH, Balabanova D. Global Mapping of Health Policy and Systems Research Training The Teaching and Learning Health Policy and Systems Research Thematic Working Group: Health Systems Global with support of the Alliance for Health Policy and Systems Research, 2015. At http://bit.ly/1N3a3jD. Accessed Apr 2016.

[33] Castells M. Information Technology, Globalization and Social Development. United nations research institute for social development (UNRISD), 1999

[34] Frenk J, Chen L, Bhutta ZA, Cohen J, Crisp N, Evans T (2010). Health professionals for a new century: transforming education to strengthen health systems in an interdependent world. Lancet.

[35] Irish Aid Department of Foreign Affairs. Programme of Strategic Cooperation between Irish Aid and Higher Education and Research Institutes 2007-2011. 2007. http://bit.ly/1bAtkYr. Accessed December 8th 2015.

[36] Downes S. e-Learning 2.0. eLearn Magazine: Education and Technology in perspective. 2005;October. At http://bit.ly/1qYiliA. Accessed Apr 2016.

[37] Virkus S (2008). Use of Web 2.0 technologies in LIS education: experiences at Tallinn University, Estonia. Program.

[38] OECD (2001). OECD E-learning: The Partnership Challenge.

[39] Johnson L, Adams S, Cummins M (2012). The NMC Horizon Report: 2012.

[40] Creswell JW, Plano-Clark VL. Designing and conducting mixed methods research. 2nd ed: Sage Publications (England); 2011.

[41] Conole G, Beetham H, Sharpe R (2013). Tools and Resources to Guide Practice. Rethinking Pedagogy for a Digital Age designing for 21st century learning.

[42] Georgina DA, Hosford CC (2009). Higher education faculty perceptions on technology integration and training. Teach Teach Educ.

[43] Vohra RS, Hallissey MT (2015). Social Networks, SocialMedia, and Innovating Surgical Education. JAMA Surgery.

[44] Woo Y, Reeves TC (2007). Meaningful interaction in web-based learning: A social constructivist interpretation. Int High Educ.

[45] Donnelly R (2010). Harmonizing technology with interaction in blended problem-based learning. Comput Educ.

[46] Song L, Singleton E, Hill J, Koh M (2004). Improving online learning: student perceptions of useful and challenging characteristics. Int High Educ.

[47] Young S, Bruce M (2011). Classroom community and student engagement in online courses. J Online Learn Teach.

[48] Weilbach L, Byrne E (2010). A human environmentalist approach to diffusion in ICT policies. A case study of the FOSS policy of the South African Government. J Info Commun Ethics.

[49] Avgerou C (2001). The significance of context in information systems and organizational change. Inf Syst J.

[50] Gedik H, Kiraz E, Ozden MY (2013). Design of a blended learning environment: Considerations and implementation issues. Australas J Educ Technol.

[51] Uzuner S. Questions of culture in distance learning: a research review. Int Rev Res Open Distance Learn. 2009;10(3).

[52] Zhao Y, Lei J, Yan B, Lai C, Tan HS (2005). What makes the difference? A practical analysis of research on the effectiveness of distance education. Teach Coll Rec.

[53] White C, Gruppen L, Dornan T, Mann K, Scherpbier A, Spencer J (2011). Identifying learners’ needs and self-assessment. Medical education theory and practice.

[54] Callon M, Law J (1989). On the construction of socio-technical networks: content and context revisited. Knowl Soc.

[55] Giddens A (1984). The Constitution of Society. Outline of the theory of structuration.

[56] Du Plooy NF. The Social Responsibility of Information Systems Developers. In: Steve C, Elayne C, Gordon MH, Andrew W, editors. Socio-Technical and Human Cognition Elements of Information Systems: IGI Publishing (London); 2003. p. 41-59

